# Arousal-Biased Competition Explains Reduced Distraction by Reward Cues under Threat

**DOI:** 10.1523/ENEURO.0099-20.2020

**Published:** 2020-07-07

**Authors:** Andy J. Kim, Brian A. Anderson

**Affiliations:** Texas A&M Institute for Neuroscience, Department of Psychological & Brain Sciences, Texas A&M University, 4235 TAMU, College Station, TX, 77843

**Keywords:** anxiety, fMRI, threat, value-driven attention

## Abstract

Anxiety is an adaptive neural state that promotes rapid responses under heightened vigilance when survival is threatened. Anxiety has consistently been found to potentiate the attentional processing of physically salient stimuli. However, a recent study demonstrated that a threat manipulation reduces attentional capture by reward-associated stimuli, suggesting a more complex relationship between anxiety and the control of attention. The mechanisms by which threat can reduce the distracting quality of stimuli are unknown. In this study, using functional magnetic resonance imaging (fMRI) on human subjects, we examined the neural correlates of attention to previously reward-associated stimuli with and without the threat of unpredictable electric shock. We replicate enhanced distractor-evoked activity throughout the value-driven attention network (VDAN) in addition to enhanced stimulus-evoked activity generally under threat. Importantly, these two factors interacted such that the representation of previously reward-associated distractors was particularly pronounced under threat. Our results from neuroimaging fit well with the principle of arousal-biased competition (ABC), although such effects are typically associated with behavioral measures of increased attention to stimuli that already possess elevated attentional priority. The findings of our study suggest that ABC can be leveraged to support more efficient ignoring of reward cues, revealing new insights into the functional significance of ABC as a mechanism of attentional control, and provide a mechanistic explanation of how threat reduces attention to irrelevant reward information.

## Significance Statement

Anxiety disorders are the most common mental illnesses in the United States. Anxiety affects how we direct our attention, which plays an important role in both adaptive and maladaptive responses to threat, but our understanding of the mechanisms underlying this relationship is limited. Here, we used neuroimaging to explore the mechanisms by which threat modulates attention to reward-related stimuli. We find that experimentally-induced anxiety interacts with the neural network associated with attentional processing of valuable stimuli, enhancing the strength with which such stimuli are represented, but behaviorally results in a reduced tendency to look at these stimuli. Our findings reveal a novel relationship between threat and attention in which enhanced stimulus-evoked activity under threat can be leveraged to facilitate ignoring.

## Introduction

Attention is a selective cognitive process that filters and prioritizes sensory information to ensure that pertinent stimuli are more strongly represented (Desimone and Duncan, 1995). Attention can be voluntarily directed to objects (Duncan and Humphreys, 1989; Corbetta and Shulman, 2002) and spatial locations (Posner, 1980; Abrams et al., 2010). In addition, attention can be biased to prioritize the processing of features that are aligned with task goals (Wolfe et al., 1989; Folk et al., 1992), but also involuntarily captured by physically salient stimuli (Theeuwes, 1992) as well as stimuli previously associated with valent outcomes including punishment (Schmidt et al., 2015; Nissens et al., 2017; Anderson and Britton, 2019c) and reward (Hickey et al., 2010; Anderson et al., 2011; Anderson, 2016).

The allocation of attention can be influenced by current emotional state, including anxiety. Anxiety is an emotional response to unpredictable threat, instilling an adaptive state of arousal and hypervigilance (Davis et al., 2010). Anxiety disorders are the most common mental illness in the United States (Kessler et al., 2005; Collins et al., 2011) and extended arousal is a hallmark of most anxiety disorders (American Psychiatric Association, 2013). Clinically anxious patients and individuals with high trait-anxiety both show increased attentional biases toward threat-related stimuli (for a meta-analysis, see Bar-Haim et al., 2007). Several theoretical frameworks have modeled the impact of anxiety on attention and cognitive performance based on the availability of limited resources, such as the dual competition model (Pessoa, 2009) and attentional control theory (Eysenck et al., 2007), in addition to attention narrowing models (Easterbrook, 1959). The literature, however, paints a complicated picture, with threat and anxiety at times facilitating and at times hindering performance across a variety of cognitive tasks (Miu et al., 2008; Grillon and Charney, 2011; Robinson et al., 2011, 2013; Cornwell et al., 2012; Hu et al., 2012; Lindström and Bohlin, 2012; Vytal et al., 2013; Yang et al., 2018). How anxiety influences information processing across different attention networks and eliciting stimuli (de Fockert et al., 2004; Corbetta et al., 2008; Shulman et al., 2009; Anderson et al., 2014; Anderson, 2019) is not well understood.

The translational threat of shock (ToS) paradigm has become a well-validated method to experimentally induce anxiety (Davis et al., 2010; Robinson et al., 2011, 2014, 2015; Schmitz and Grillon, 2012). Recently, the ToS paradigm was applied to evaluate how anxiety modulates attentional biases to different types of stimuli. It was found that the threat of random, unpredictable electric shock increases susceptibility to attentional capture by physically salient stimuli (Kim and Anderson, 2019c), consistent with previous findings from individuals with high trait-anxiety (Moser et al., 2012; Esterman et al., 2013) and the principle of arousal-biased competition (ABC) by which negative arousal (heightened arousal evoked by a negatively-valenced event or state) biases attention more strongly toward already high-priority stimuli (Lee et al., 2012, 2014; Sutherland and Mather, 2012, 2015). In contrast, however, ToS was found to reduce attentional capture by previously reward-associated stimuli (Kim and Anderson, 2019c). These findings indicate that there may be a fundamental mechanistic difference in how anxiety modulates different attention networks, with enhanced processing of physically salient stimuli but blunted processing of reward-related stimuli.

In this study, we used functional magnetic resonance imaging (fMRI) to probe the modulatory influence of threat on the neural representation of former targets that were previously associated with reward. Participants first completed a training phase in which a color-defined target was paired with high reward. In the subsequent test phase, we measured the influence of this training on eye movements and stimulus-evoked responses in the brain, both with and without the concurrent ToS. We hypothesized an interaction in behavior by which oculomotor capture by the previously reward-associated former-target (distractor) is reduced under ToS, replicating previous results (Kim and Anderson, 2019c). Also consistent with prior results, we predicted elevated distractor-evoked responses in regions of the brain previously linked to value-driven attention, including the value-driven attention network (VDAN): extrastriate visual cortex, frontal eye field (FEF), intraparietal sulcus (IPS), and caudate tail (Anderson et al., 2014, 2017; Anderson, 2017; Kim and Anderson, 2020), in addition to the insula (Wang et al., 2015), ventral striatum (Meffert et al., 2018), and amygdala (Peck and Salzman, 2014; Ousdal et al., 2014). We further hypothesized that the ToS would be associated with increased stimulus-evoked responses in these regions, reflecting a global effect of arousal on visual information processing. Of particular interest in the present study was the interaction between distractor-evoked neural responses and threat. The dual competition framework (Pessoa, 2009) predicts reduced distractor-evoked responses under threat, mirroring the hypothesized pattern in behavior. In contrast, the ABC model (Mather and Sutherland, 2011) predicts elevated distractor-evoked activity under threat, consistent with the influence of negative arousal on the processing of physically salient stimuli (Lee et al., 2014). Given the intuitive fit between the dual competition framework (Pessoa, 2009) and previously observed behavioral results (Kim and Anderson, 2019c), we hypothesized reduced distractor-evoked responses under threat.

## Materials and Methods

### Participants

Forty-one participants were recruited from the university community. All participants were English speaking and reported normal or corrected-to-normal visual acuity and normal color vision. Four participants withdrew from the experiment before completing the brain scans and one participant was not scanned because they did not meet the performance criteria for the behavioral task during their initial in-lab visit. Thus, 36 participants were fully scanned (18 female, ages 18–35; mean = 22.9 years, SD = 4.33 years), and eye-tracking data were collected from 27 of these participants (due to eye-tracker availability and technical difficulties in the scanning environment).

### Ethics statement

All procedures were approved by the university Institutional Review Board and were conducted in accordance with the principles expressed in the Declaration of Helsinki. Written informed consent was obtained for each participant.

### Task procedure

Participants were scheduled for an initial in-lab visit for 1 h, and each eligible participant underwent fMRI in a single 1.5-h session at the scan-center on the following day. During their initial appointment, participants came into the lab for consenting, MRI safety screening, screening for adequate performance on the behavioral tasks, and familiarization with the shock delivery protocol. Participants first completed the test phase task once under the ToS (to familiarize them with the task procedure without interfering with prior learning) and then the training phase task three times to establish learning of the stimulus-reward associations. During the fMRI session, participants completed two runs of the training phase and the test phase, an anatomic scan, and an additional two runs of the training phase and the test phase. One pair of test phase runs was performed under ToS (see below, Design) and is referred to as the threat block. Two runs of reward training were completed before each block of the test phase to mitigate possible extinction effects between the two blocks. Before entering the scanner, participants underwent a shock calibration procedure to achieve a level of shock that is “unpleasant, but not painful” (Anderson and Britton, 2019c; Kim and Anderson, 2019c) and were then disconnected from the shock device. Participants were reconnected to the shock device before beginning the test phase of the threat block and were immediately disconnected from the device after completion of the threat block. The anatomic scan was inserted after the first test phase to allow for the anxiety-inducing nature of the shock device to dissipate in participants who completed the threat block first, as seen in within-subject designs of the ToS paradigm (Kim and Anderson, 2019c). Participants were compensated the total monetary reward accumulated at the end of the last training phase or the combined amount of $10/h spent in the initial appointment session and $20/h spent in the fMRI session, whichever amount was higher.

### Apparatus

During the initial in-lab visit, all tasks were completed on a Dell OptiPlex 7040 computer (Dell) equipped with MATLAB software (MathWorks), and Psychophysics Toolbox extensions (Brainard, 1997). Stimuli were presented on a Dell P2717H monitor. The participants viewed the monitor from a distance of ∼70 cm in a dimly lit room. Paired electrodes (BioPac Systems) were attached to the left forearm of each participant, and electric shocks were delivered through an isolated linear stimulator under the constant current setting (STMISOLA, BioPac Systems), which were controlled by custom MATLAB scripts.

For the fMRI portion of the experiment, stimulus presentation was controlled by an *in vivo* SensaVue display system. The eye-to-screen distance was ∼125 cm. Key responses were entered using Cedrus Lumina two-button response pads. MRI-compatible electrodes (BioPac Systems) were attached to the left ankle of each participant, and electric shocks was delivered through an STM100C controlled by an MP160 system (BioPac Systems) triggered by custom MATLAB scripts via parallel port interface. An EyeLink 1000 Plus system was used to track eye position (SR Research Ltd.).

### Design

We adopted the design of experiment 3 in Kim and Anderson (2019c) with modifications for fMRI. Both the training and test phases were split into two runs, with each run consisting of 60 trials. In the test phase, the order of threat block first or no-threat block first was counterbalanced across participants. In each run of the threat block, participants were shocked two, three, or four times every 20 trials (order randomized) for a total of nine times during the entire run. The pattern of shocks administered in the threat block across trials was pseudo-randomly determined with the constraint that shocks were never administered on consecutive trials nor on the last trial of a run. At the end of the experiment, participants were paid the total monetary reward obtained during the training phase (spanning both the in-lab and in-scanner portions of the experiment).

### Training phase

**Figure 1.**
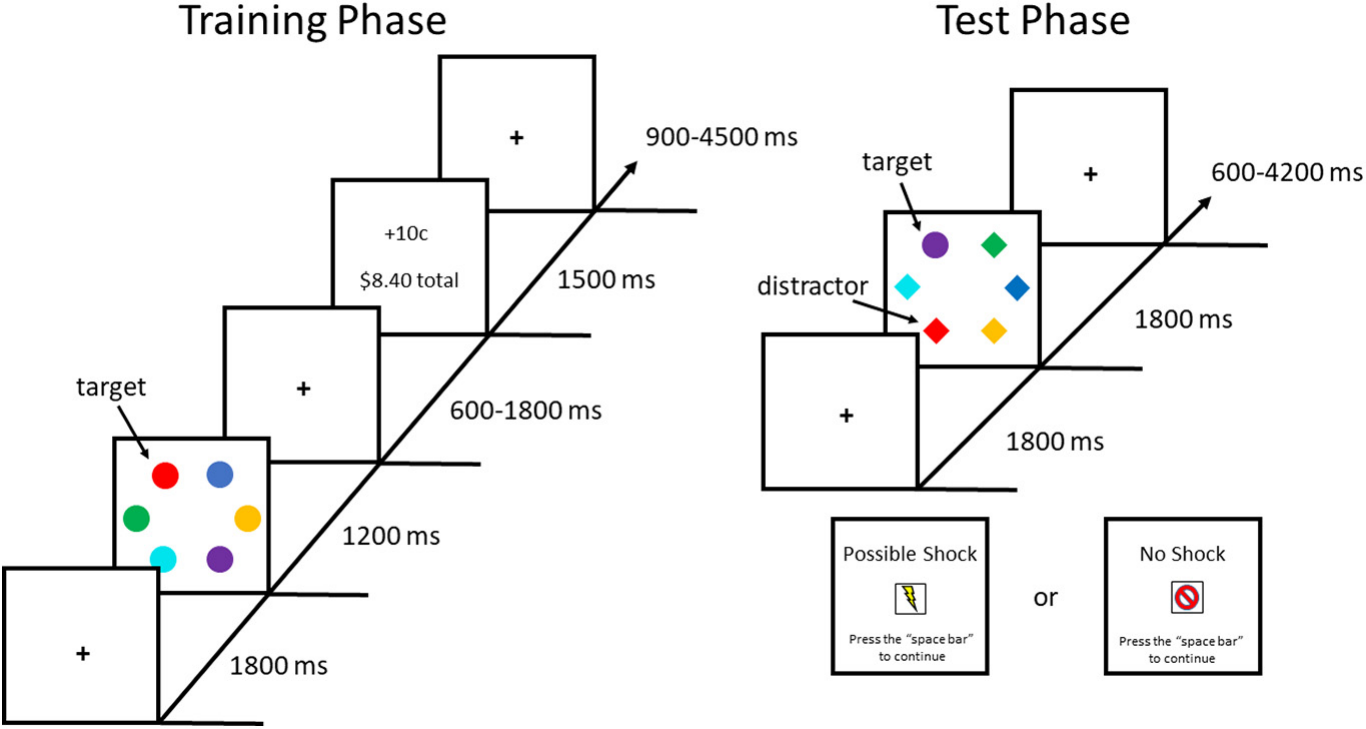
Sequence of trial events. In the training phase, participants searched for a target defined by color (red or green, one that was present on each trial), and correct responses were followed by the delivery of monetary reward feedback. In the test phase, participants searched for a target defined as the unique shape, and no reward feedback was provided. Half of the trials contained the previously rewarded color as a non-target distractor. The test phase was split into the threat and no-threat blocks, in which it was possible to receive unpredictable electric shocks or no chance of receiving shock, respectively.

In the training phase, each trial began with a fixation display (1800 ms), followed by a search array (1200 ms), an interstimulus interval (ISI) consisting of a fixation cross, a reward feedback display (1500 ms), and an intertrial interval (ITI; Fig. 1). The fixation display consisted of a fixation cross (0.7° × 0.7° visual angle) at the center of the screen. The search array consisted of six colored circles, three on each side of the display. During the search array, participants were instructed to search for a target circle that was unpredictably red or green (each target color appeared equally-often) and report the identity of the letter inside of the target as X or V using the response pad. Letters inside the non-targets were randomly assigned from the pool of H, Y, L, N, and K (without replacement). The letter-report procedure was used to require foveation of the target (Theeuwes et al., 1998, 1999), as not all participants could be tracked with the eye tracker, precluding the use of an explicitly gaze-contingent task (in which the only response was an eye movement) as in Kim and Anderson (2019c). Each target color appeared at every position equally-often across trials and the order of trials was randomized for each run. Each circle in the search array was 4.5° visual angle in diameter. Stimuli located on the left and right sides were 8.2° (upper and lower positions) and 10.6° (center positions) visual angle from the meridian. Vertically, stimuli appearing in the upper and lower positions were 8.2° visual angle above and below the horizontal equator. The colors of the non-targets were drawn from the set [blue, cyan, pink, orange, yellow, white] without replacement. The ISI lasted for 600, 1200, or 1800 ms (equally-often). For each participant, one of the color targets (counterbalanced) would yield a monetary reward of 25¢ on 80% of trials and 5¢ on 20% of trials (high-value target); the other color target would yield 5¢ on 80% of trials and 25¢ on 20% of trials (low-value target). Lastly, the ITI lasted for 900, 2700, or 4500 ms (exponentially distributed with the shortest time being the most frequent). The fixation cross disappeared for the last 200 ms of the ITI to indicate to the participant that the next trial was about to begin.

### Test phase

In the test phase, each trial began with a fixation display (1800 ms), followed by a search array (1800 ms) and an ITI (Fig. 1). The fixation display was identical to that of the training phase. During the search array, participants looked for the uniquely-shaped target and performed the same letter-judgment task on the target. The color of the shapes was irrelevant to the task. On half of the trials, one of the non-target shapes was rendered in the color of the former high-value target during the training phase (referred to as the distractor). The other half of trials did not contain either of the prior target colors from training (distractor-absent trials); the low-value color did not appear during the test phase to maximize the trials-per-cell in the factorial design (as in Kim and Anderson, 2019c). The target was equally-often a diamond among circles and a circle among diamonds, and was never red or green. The target appeared on each side of the screen equally-often for both distractor-present and distractor-absent trials, and on distractor-present trials the side of the distractor was unbiased with respect to the side of the target (2/5 same side, 3/5 opposite side, corresponding to five stimulus positions not occupied by the target). The size and positions of the stimuli were identical to the training phase, as was the set of non-target colors used. Lastly, the ITI lasted for 600, 2400, or 4200 ms (equally-often). The fixation cross disappeared for the last 200 ms of the ITI to indicate to the participant that the next trial was about to begin. On trials in which a shock was delivered, an additional “pseudo-trial” was inserted and shock was administered after the fixation display in place of the search array, followed by the ITI. In the no-threat block, pseudo-trials were also included but without shock administration to maintain the timing and flow of the trials across blocks.

### Eye-tracking

During the fMRI scan, head position was restricted using foam padding within the head coil, and eye-tracking was conducted using the reflection of the participant’s face on the mirror attached to the head coil. Eye position was calibrated before each run of trials using nine-point calibration (Anderson and Yantis, 2012; Kim and Anderson, 2020) and was manually drift corrected by the experimenter as necessary during the fixation display. As the modulatory influence of threat on attentional capture by previously reward-associated stimuli was previously observed in distractor-evoked eye movements (Kim and Anderson, 2019c), we sought to replicate this behavioral effect by measuring eye position in the present study.

### Analysis of eye-tracking data

Following each run, recorded fixation events were analyzed off-line using custom MATLAB scripts. Fixations within a 6.3° window centered on and extending beyond the boundary of a stimulus, made during the period of time that the search array was on the screen, were attributed to that stimulus. The window size was chosen to roughly maximize the margin for error in measured eye position without creating ambiguity in which stimulus was fixated. Fixations were analyzed using the output file from the EyeLink host computer, in which saccades were defined as occurring when velocity exceeded 35°/s and acceleration exceeded 9500°/s^2^ (Anderson and Yantis, 2012; Anderson and Kim, 2018a,b). We measured which of the six shape stimuli in the test phase was initially fixated on each trial (i.e., the first of the six stimuli fixated). Oculomotor capture was determined by comparing the probability of initially fixating the high-value distractor (number of trials on which the high-value distractor was fixated/all trials on which a high-value distractor appeared) compared with the average of other non-target stimuli (i.e., corrected for the number of non-targets present in the display). We focused our analyses on oculomotor capture, rather than saccadic reaction time (RT), given its superior reliability (Anderson and Kim, 2019b; see also Anderson and Kim, 2019a; Anderson et al., 2019) and its relation to threat-based modulation in prior research (Kim and Anderson, 2019c).

The influence of threat on oculomotor capture was assessed by means of a 2 × 2 ANOVA with reward association (high-value distractor vs other non-target) and block (threat vs no threat) as factors. In the event of the hypothesized interaction, the nature of the interaction would be probed by comparing eye movements across blocks separately for the high-value distractor and other non-targets, to determine whether threat-related changes in fixations were specific to fixations on the high-value distractor or whether the accuracy of eye movements was affected more broadly (including eye movements to non-targets other than the high-value distractor).

Lastly, to verify whether our ToS modulation induced a state of heightened negative arousal in the threat block as intended, we compared pupil size between the threat and no-threat blocks as an indicator of arousal (Bradley et al., 2008; Nassar et al., 2012). Specifically, mean pupil size was measured during the 1800-ms fixation period at the beginning of each trial, averaged across all trials separately for each block, and then compared between blocks using Student’s *t* test. Furthermore, we correlated the difference in mean pupil size between blocks with the interaction term corresponding to oculomotor capture (difference in the difference scores from the above 2 × 2 ANOVA) to determine whether the magnitude of negative arousal as measured from pupil size was related to the influence of threat on oculomotor capture.

### MRI data acquisition

Images were acquired using a Siemens 3-Tesla MAGNETOM Verio scanner with a 32-channel head coil. High-resolution whole-brain anatomic images were acquired using a T1-weighted magnetization prepared rapid gradient echo (MPRAGE) pulse sequence [150 coronal slices, voxel size = 1 mm isotropic, repetition time (TR) = 7.9 ms, echo time (TE) = 3.65 ms, flip angle = 8°]. Whole-brain functional images were acquired using a T2*-weighted echoplanar imaging (EPI) multiband pulse sequence (56 axial slices, TR = 600 ms, TE = 29 ms, flip angle = 52°, image matrix = 96 × 96, field of view = 240 mm, slice thickness = 2.5 mm with no gap). Each EPI pulse sequence began with dummy pulses to allow the MR signal to reach steady state and concluded with an additional 6-s blank epoch. Each run of the training phase lasted 504 s and each run of the test phase (for both the threat and no-threat block) lasted 428.4 s (including dummy pulses).

### MRI data analyses

#### Preprocessing

All preprocessing was conducted using the AFNI software package (Cox, 1996). Each EPI run for each participant was motion corrected using 3dvolreg, using the first image following the anatomic scan as a reference. The anatomic image was skull-stripped using 3dskullstrip and non-linearly registered to the Talairach brain (Talairach and Tournoux, 1988) using auto_warp.py. EPI images were coregistered to the corresponding anatomic image for each participant using align_epi_anat.py, and the EPI then converted to percent signal change normalized to the mean of each run. Lastly, EPI images were non-linearly warped to the Talairach brain by applying the warp parameters from the anatomic image using 3dQwarp, and then spatially smoothed to a resulting 5-mm full-width half-maximum smoothness using 3dBlurToFWHM.

### Statistical analyses

All statistical analyses were performed using the AFNI software package (Cox, 1996). We used a general linear model (GLM) approach to analyze the test phase data. The test phase was split into the threat and no-threat blocks and a separate GLM was conducted on each. Each GLM included the following task-based regressors: (1) target on left, distractor on same side; (2) target on left, distractor on opposite side; (3) target on right, distractor on same side; (4) target on right, distractor on opposite side; (5) target on left, no distractor; and (6) target on right, no distractor. The hemifield in which the stimuli appeared was included in the model in keeping with prior studies of value-driven attention, as some distractor-evoked activity is known to be modulated by this factor (Anderson et al., 2014; Anderson, 2019; Kim and Anderson, 2019a, 2020). Experience of shock (or the absence of shock on pseudo trials in the no-threat block) was included as a regressor of non-interest. Each of these regressors was modeled using 16 finite impulse response functions (FIRs), beginning at stimulus onset (Kim and Anderson, 2020; see also Kim and Anderson, 2019b). Six degrees of head motion and drift in the scanner signal were modeled using nuisance regressors. Trials in which the participant failed to make a motor response were excluded from the analyses.

The peak β value for each task-based regressor from 3 to 6 s post-stimulus presentation was extracted (Kim and Anderson, 2019b, 2020). We first looked for regions sensitive to both the reward and the threat manipulation, which would serve as candidate regions for threat-based modulation of distractor processing. To this end, we computed the intersection of the effects of distractor condition and threat. The main effect of threat was determined by contrasting task-based regressors corresponding to the threat versus no-threat blocks. The effect of distractor condition was determined separately for each combination of distractor and target position with the effect of target position factored out. Specifically, we contrasted task-based regressors (1) 1 versus 5, (2) 2 versus 5, (3) 3 versus 6, and (4) 4 versus 6, collapsing across regressors corresponding to threat and no-threat blocks. This was done to preserve information about the position of the distractor, which is known to affect neural responses in the visual system (Anderson et al., 2014; Anderson, 2017, 2019; Kim and Anderson, 2019a). The results from each contrast were corrected for multiple comparisons using the AFNI program 3dClustSim, with the smoothness of the data estimated using the ACF method (clusterwise α < 0.05, voxelwise *p *<* *0.005). Significant clusters of activation for each individual contrast were identified, and regions of overlap between each distractor contrast and the main effect of threat were determined (intersection of the respective activation maps), and then collapsed across the four contrasts to determine the entire extent of overlap.

Next, we probed for interactions between distractor condition and threat within regions identified in the prior analysis (i.e., clusters of voxels in which both an effect of distractor and threat were identified) using a region of interest (ROI) approach, which served as our primary analysis of interest that would discriminate between the competing predictions outlined in the Introduction. Since the regions of the VDAN are well-established to play an integrated role in the value-driven control of attention (Anderson et al., 2014, 2017; Anderson, 2017, 2019; Kim and Anderson, 2020), we planned a priori to collapse across any regions identified within this network for this analysis and, along with any of the other regions previously implicated in value-driven attention as outlined in our hypothesis (see Introduction), apply Bonferroni correction for multiple comparisons. We used an ROI approach with a leave-one-subject-out procedure to preserve independence (Esterman et al., 2010) so that we could extract conditional means (Anderson et al., 2016a) to examine the specific nature of the interaction (i.e., assess directionality). To this end, we extracted per-region conditional means from distractor-present and distractor-absent trials, separately for the threat and no-threat blocks using the AFNI program 3dmaskave, and then computed the interaction term for these conditional means via a 2 × 2 within-subjects ANOVA (computed in SPSS). This interaction analysis focused specifically on distractor-present trials where the target and distractor were presented in opposite hemifields (task-based regressors 2 and 4), to better isolate task-irrelevant information processing in keeping with prior studies on the neural correlates of value-driven attention (Anderson et al., 2014; Anderson, 2017; Kim and Anderson, 2019a,c).

Finally, to assess potential links between the behavioral effect of threat on distractor-evoked eye movements and brain activation, we entered the difference between the frequency of fixations on the critical distractor in no-threat and threat blocks as a covariate in a contrast comparing activation on distractor-present trials (collapsing across the four combinations of target and distractor position) between the no-threat and threat blocks. The interaction between distractor condition and the covariate was set up such that a significantly positive relationship would indicate that more blunted oculomotor capture by threat was associated with more reduced distractor-evoked activity under threat and a significantly negative relationship would indicate the opposite. This covariate analysis was corrected for multiple comparisons at the cluster level in the same manner as the other contrasts as described above.

### Data availability statement

All anonymized study data, including the raw MRI data, are freely available on the Open Science Framework (https://osf.io/rk6p4/). Data sharing for this article complies with the requirements of the funding agencies and the stipulations of the university IRB approvals.

## Results

### Behavior

During the training phase, eye movements were recorded to one of the six shape stimuli on 90.2% of trials (SD = 10.4%). On trials in which a fixation was made and the high-value color was the target, first fixations to the target were made 58.7% of the time (SD = 17.3%) and on low-value target trials, first fixations to the target were made 58.6% of the time (SD = 14.6%). The likelihood of the first fixation falling on the target did not differ with respect to target value, *t*_(26)_ = 0.11, *p = *0.910.

**Figure 2.**
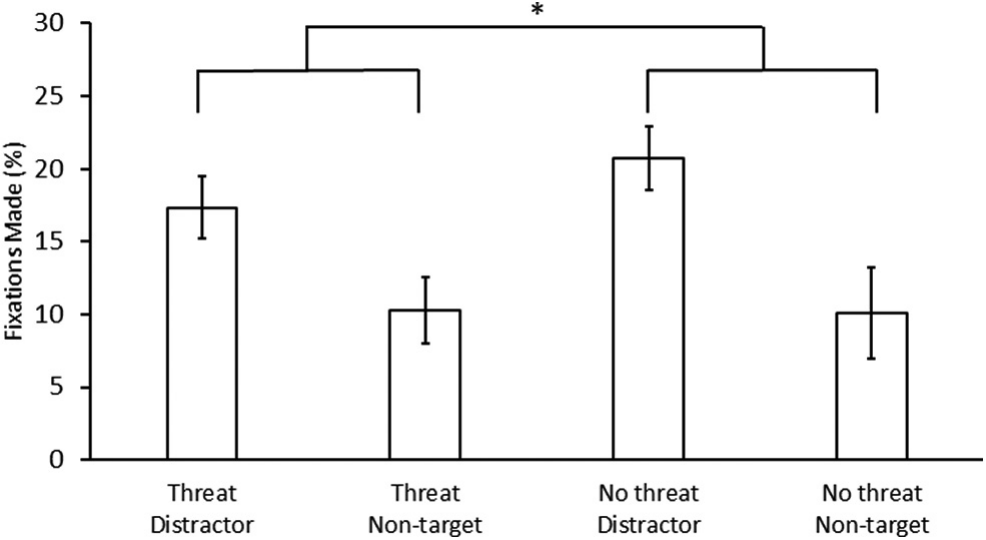
Oculomotor capture in the test phase. Data are broken down by block (threat vs no-threat) and first fixations made each trial on the previously reward-associated distractor versus a non-target. Error bars depict within-subject CIs calculated using the Cousineau method with a Morey correction; *p <* *0.05.

During the test phase, eye movements were recorded on 82.0% of trials (SD = 15.1%). During the threat block, first fixations to the distractor and non-target shapes occurred on average 17.3% (SD = 7.6%) and 10.3% (SD = 4.6%) of trials, respectively. During the no-threat block, first fixations to the distractor and non-target shapes occurred on average 20.7% (SD = 10.3%) and 10.1% (SD = 4.4%) of trials, respectively. There was a significant main effect of reward association, *F*_(1,26)_ = 25.03, *p *<* *0.001, η^2^ = 0.490, 95% confidence interval (CI) [5.38, 12.32], main effect of block, *F*_(1,26)_ = 5.09, *p *=* *0.033, η^2^ = 0.164, 95% CI [0.21, 2.97], and interaction between reward association and block with attentional capture being reduced under ToS, *F*_(1,26)_ = 4.94, *p *=* *0.035, η^2^ = 0.160, 95% CI [0.42, 6.74] (Fig. 2), replicating the pattern of performance observed in Kim and Anderson (2019c). Probing the interaction, fixations on the critical distractor differed between the threat and no-threat block, *t*_(26)_ = 2.32, *p *=* *0.029, *d *=* *0.45, 95% CI [0.38, 6.37], but fixations on non-targets did not, *t*_(26)_ = −0.50, *p *=* *0.624.

### Pupil size

Measured pupil size was larger during the fixation period leading up to presentation of the stimulus array in the threat compared with the no-threat block, *t*_(26)_ = 2.56, *p *=* *0.016, *d *=* *0.49, 95% CI [21.06, 191.55], confirming the arousing nature of the threat manipulation. The correlation between this threat effect and the interaction term from the ANOVA on fixations was marginally significant, *r *=* *0.331, *p *=* *0.09.

### Neuroimaging

**Figure 3.**
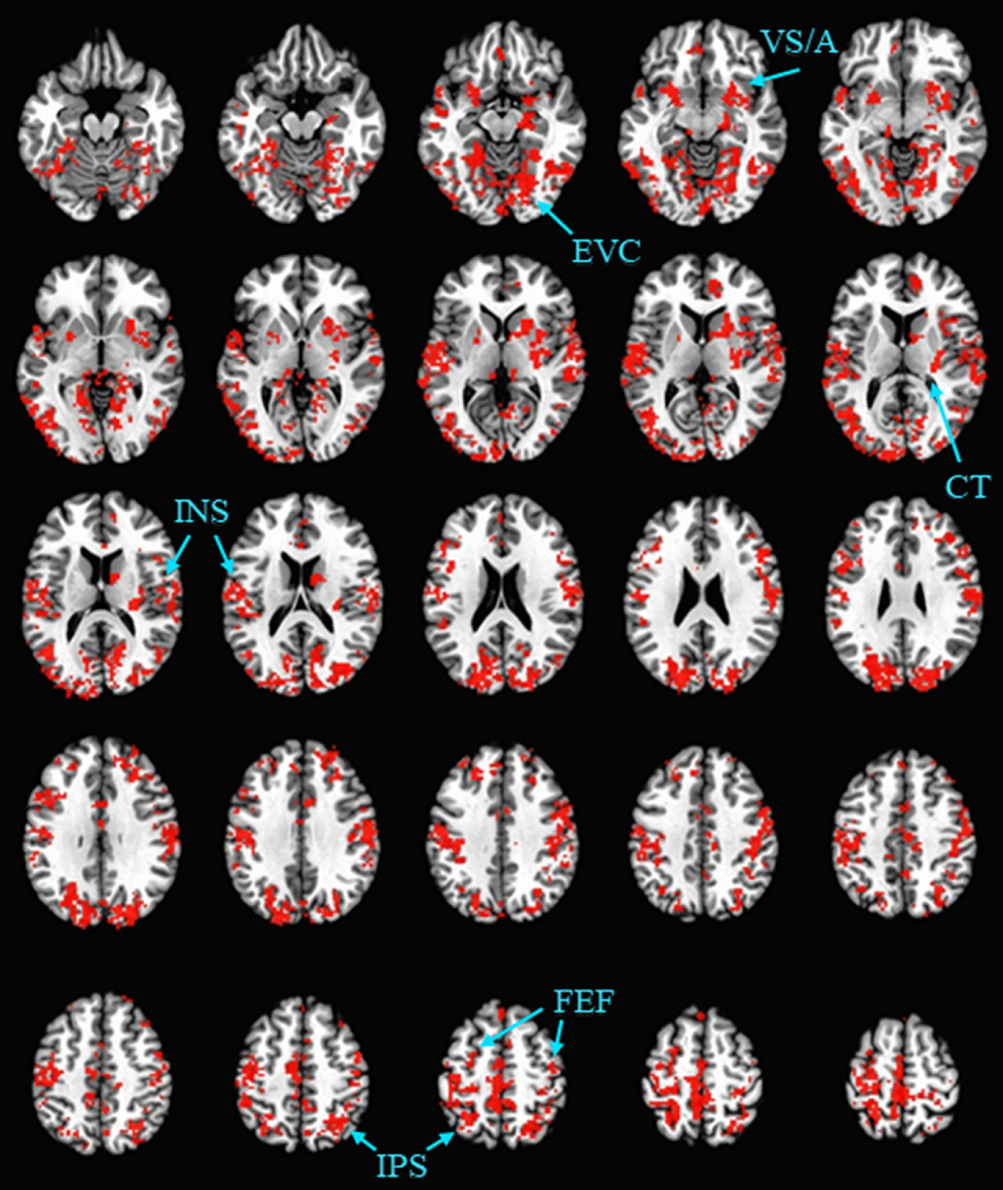
Montage of regions for which both an effect of threat and distractor condition were evident. Significant clusters were identified for each effect (clusterwise α < 0.05, voxelwise *p *<* *0.005) and the intersection of the resulting activation maps was computed and used for ROI definition (ROIs indicated with the labels and arrows). The intersection is shown across subjects using the leave-one-subject-out procedure to depict the full spatial extent of the ROIs used. The resulting activations are overlaid on an image of the Talairach brain. See Extended Data Figure 3-1 for activation masks for each main effect. EVC, extrastriate visual cortex; VS/A, ventral striatum/amygdala; INS, insula; CT, caudate tail; FEF, frontal eye fields; IPS, intraparietal sulcus.

First, we were interested in regions in which stimulus processing was sensitive to both the distractor and the threat manipulation. To this end, we computed the intersection of the effect of distractor and threat (see Materials and Methods). Each of the hypothesized regions were identified in this analysis, including the extrastriate visual cortex, FEF, IPS, and caudate tail (collectively, the VDAN), in addition to the insula, ventral striatum, and amygdala (Fig. 3; Extended Data Fig. 3-1).

10.1523/ENEURO.0099-20.2020.f3-1 Extended Data Figure 3-1 Effect of distractor (green), threat (orange), and overlap between the two (red) depicted for each of the four distractor contrasts: (***A***) distractor on left, target on opposite side, (***B***) distractor on right, target on opposite side, (***C***) distractor on right, target on same side, and (***D***) distractor on left, target on same side. Note that these activation maps are shown with all subjects included, the contrasts underlying which served as the basis for the leave-one-subject-out procedure for ROI definition (with the full extent of the resulting ROIs shown across subjects in Fig. 3). Download Figure 3-1, TIF file.

**Figure 4.**
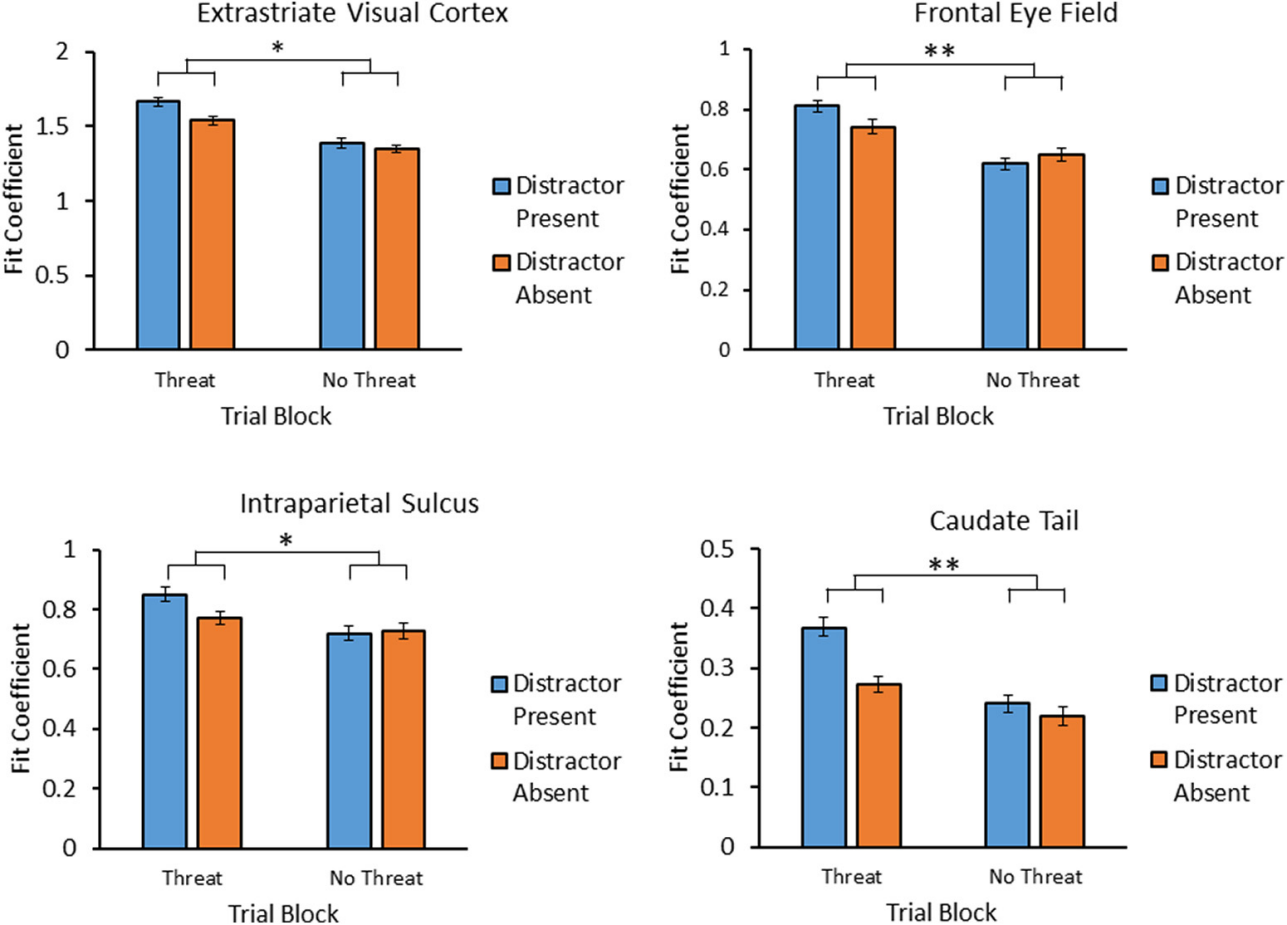
Interaction of threat (threat vs no-threat) and distractor condition (present vs absent) in the extrastriate visual cortex, FEF, IPS, and caudate tail. Error bars depict within-subject CIs calculated using the Cousineau method with a Morey correction; *p <* *0.05, **p <* *0.01.

**Figure 5.**
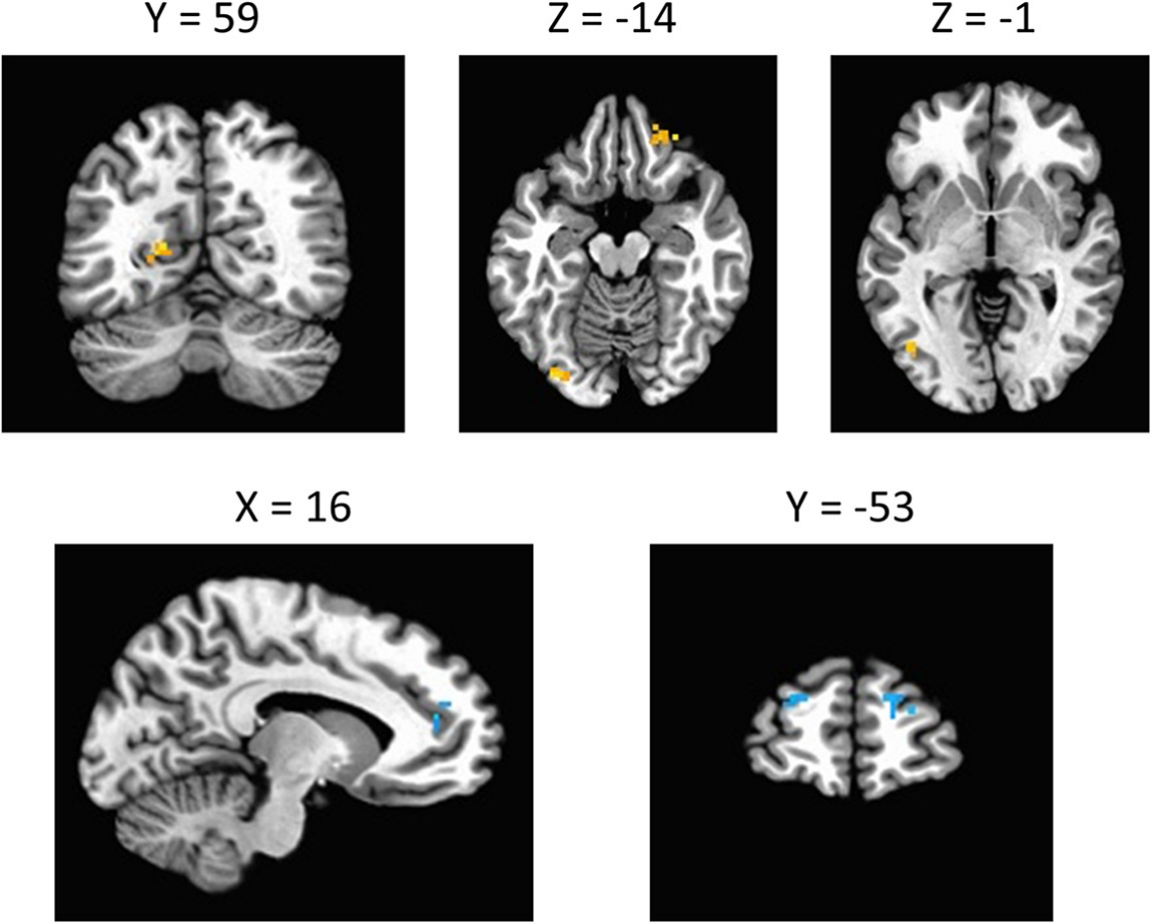
Significant clusters, overlaid on an image of the Talairach brain, where the modulation of distractor-evoked brain activity by threat was related to the influence of threat on oculomotor capture.

We next tested for an interaction between value (distractor condition) and block within the aforementioned regions, which served as ROIs in a follow-up contrast (see Materials and Methods). First, focusing specifically on the regions of the VDAN, the interaction was significant, *F*_(1,35)_ = 8.74, *p *=* *0.006, η^2^ = 0.200, 95% CI [0.02, 0.13], which was sufficiently robust to pass correction for multiple comparisons (see Materials and Methods). Further probe of the interaction within the VDAN revealed that the interaction was individually significant within each region of the VDAN, attesting to the assumption that they form an integrated network (see Table 1). Surprisingly, the direction of this interaction was opposite that of the behavioral interaction, with the distractor evoking stronger activation under threat in each individual region (Fig. 4). No reliable interaction was evident in the insula or ventral striatum/amygdala (which formed one contiguous cluster; see Table 1). As a covariate (see Materials and Methods), the reduction in distractor fixations in the threat block was associated with reduced distractor-evoked activation under threat in the orbitofrontal and visual cortex, in addition to increased distractor-evoked activation under threat in the dorsolateral prefrontal and anterior cingulate cortex (Fig. 5).

**Table 1:** Interaction effect between threat and reward-associated distractor for main ANOVA contrast conducted over voxel activation in each ROI

ROIs	*F*	*p*	η^2^	95% CI
Visual cortex	4.249	0.047	0.108	[0.004, 0.169]
FEFs	9.980	0.003	0.222	[0.04, 0.16]
IPS	6.985	0.012	0.166	[0.02, 0.16]
Caudate tail	8.041	0.008	0.187	[0.02, 0.12]
Insula	0.370	0.547	0.010	[–0.06, 0.10]
Ventral striatum + amygdala	0.645	0.428	0.019	[–0.10, 0.24]

## Discussion

In the present study, we used the value-driven attentional capture (VDAC) paradigm (Anderson et al., 2011) combined with a ToS manipulation (Davis et al., 2010; Schmitz and Grillon, 2012) to determine the neural mechanisms of reduced attentional capture by reward-associated stimuli under conditions of experimentally-induced anxiety. As in Kim and Anderson (2019c), our behavioral results reveal reduced oculomotor capture by previously reward-associated distractors under threat, and our neuroimaging data replicate the neural correlates of VDAC throughout the VDAN, including the extrastriate visual cortex, IPS, FEF, and caudate tail (Anderson et al., 2014, 2016b, 2017; Hickey and Peelen, 2015; Anderson, 2017, 2019; Kim and Anderson, 2020). We also observed elevated stimulus-evoked activity under threat, consistent with enhanced sensory processing due to negative arousal. Surprisingly, we additionally observed an interaction within the VDAN whereby reward-associated distractors evoked particularly-elevated responses under threat, counter to our hypothesis. Stronger effects of threat on distractor-evoked eye movements were associated with a more pronounced reduction in distractor-evoked activity in the orbitofrontal and visual cortex and a more pronounced increase in distractor-evoked activity in the frontal cortex, potentially reflecting a threat-related modulation of stimulus-evoked activity (O'Doherty, 2004; Anderson et al., 2014; Anderson, 2017, 2019) and cognitive control (Chao and Knight, 1995; Ochsner and Gross, 2005; Corbetta et al., 2008; Sanchez-Lopez et al., 2018), respectively. These findings suggest that reduced distractibility by reward cues under threat, as measured from behavior (eye movements), is not due to competition between positive and negative valence for limited information-processing resources, which would have predicted the opposite pattern, but are rather more aligned with the framework of ABC (Mather and Sutherland, 2011).

The ABC model is derived from theories of biased competition (Desimone and Duncan, 1995; Itti and Koch, 2000) and postulates that negative arousal biases perceptual competition in favor of already high-priority stimuli at the expense of less salient stimuli (Mather and Sutherland, 2011). That is, under states of negative arousal, the difference in the strength with which high priority and low priority stimuli are processed becomes even more pronounced. In this study, we uncover that negative arousal due to ToS increases activation within the VDAN by high-priority (by virtue of their associated value) distractors in an oculomotor task, as would be predicted by the ABC model. However, this pattern in the stimulus-evoked brain responses was associated with reduced attentional capture by the distractors as measured with eye movements, in contrast to the behavioral predictions arising from the ABC model. Our findings therefore call for a reinterpretation of the relationship between ABC and perceptual processing, at least with respect to overt attention.

Arousal has been shown to improve task performance and reduce errors (Grillon et al., 2017). Prior investigations of the ABC model have demonstrated increased attentional processing of stimuli that already possess elevated attentional priority, often operationally defined in terms of physical salience (Lee et al., 2012, 2014; Sutherland and Mather, 2015). Heightened attention to such stimuli could be considered adaptive under these circumstances, as the introduction of a physically salient stimulus could signal a potential new threat that needs to be evaluated and responded to (Esterman et al., 2013). In contrast, the previously reward-associated stimuli used in our study were not physically salient and were known to be task-irrelevant, but still possessed elevated attentional priority by virtue of their learning history. One potential interpretation of our findings is that, consistent with the ABC model, negative arousal preferentially biases stimulus representation in favor of stimuli that already have high priority. However, the influence of this bias on the orienting response is not obligatory, but rather contingent on the nature of the eliciting stimulus. If the eliciting stimulus is survival-relevant, as in the case of physically salient stimuli, it will trigger an orienting response, but if the eliciting stimulus explicitly poses no potential danger, as in the case of a previously reward-associated stimulus, observers are able to use the arousal-biased signal to “mark” the stimulus for ignoring.

The signal suppression hypothesis has been proposed as a model of attentional selection in which priority signals can be suppressed during goal-directed, feature-based visual search (Sawaki and Luck, 2010; Gaspelin and Luck, 2018b). This model has been repeatedly validated in event-related potential studies showing active suppression of a physically-salient stimulus (for a review, see Gaspelin and Luck, 2019). Furthermore, this phenomenon has been demonstrated in studies of overt attention in which the frequency of oculomotor capture by distractors is reduced via suppressive mechanisms (Ipata et al., 2006; Gaspelin et al., 2015; Gaspelin and Luck, 2018a). The neural correlates of signal suppression are not well understood and have not yet been investigated using fMRI. Our behavioral results are consistent with the concept of signal suppression under threat, although it is important to note that any threat-related suppression of distraction was only partial such that the previously high-value distractors still drew eye movements to some degree across all conditions in our study. More generally, however, our findings clearly demonstrate that elevated stimulus-evoked responses in the brain can lead to enhanced ignoring as measured from behavior, which may prove to be an important principle in understanding mechanisms of signal suppression. In this regard, it is noteworthy that signal suppression seems to be particularly effective for stimuli that evoke strong responses in the visual system by virtue of their physical salience (Gaspelin et al., 2015; Gaspelin and Luck, 2018a,b; Hickey et al., 2009), necessitating some relationship between mechanisms of suppression and elevated stimulus-evoked activity in the visual system. Our findings are also consistent with a prior report showing that parametrically increasing salience or associated value can under certain circumstances reduce the magnitude of distraction (Moher et al., 2015), further supporting the notion that suppression of behavioral distraction might at times be facilitated by strengthening the representation of a stimulus in the visual system.

Prior studies in support of the ABC model have used fear-conditioned startle reflexes as negative arousal (Sutherland and Mather, 2012; Lee et al., 2014) or negatively-valenced images (Lee et al., 2012) in the context of visual search. In our study, in contrast, negative arousal resulted from the threat of an unpredictable and aversive biological event. Both methodologies have produced results in support of the ABC model (Lee et al., 2012, 2014; Sutherland and Mather, 2012; Kim and Anderson, 2019c), but studies using startle reflexes have consistently demonstrated increased attentional capture while reduced attentional capture by reward cues has only been tested in the context of ToS. However, increased attentional capture by physically salient stimuli has been previously observed using the ToS paradigm (Kim and Anderson, 2019c), arguing that the contrasting behavioral results are not a by-product of the methodology used to induce negative arousal. At the same time, an increasingly nuanced understanding of fear- and anxiety-associated neural networks have determined fundamental differences between cognitive processing during imminent versus unpredictable threat (Davis et al., 2010), and so we restrict our conclusions to the influence of unpredictable threat.

Another way in which our study differs from prior studies supporting the ABC model is in the role of memory in the attentional priority of the distractor. In the present study, the distractors were preferentially attended by virtue of their status as previously high-value targets, which contrasts with the attentional priority of the physically salient stimuli frequently used in studies of ABC (Mather and Sutherland, 2011; Lee et al., 2012, 2014; Sutherland and Mather, 2015), which is not memory dependent. One possibility is that threat modulates access to, or the recruitment of, memory for the pertinence of stimuli, which may have impacted the influence of such memory on eye movements and/or stimulus processing in the visual system of the brain. It is also possible that our threat manipulation had a more direct impact on visual information processing, as hypothesized by the ABC model (Mather and Sutherland, 2011; Lee et al., 2012, 2014; Sutherland and Mather, 2015). Attention and memory are intricately intertwined (for review, see Chun and Turk-Browne, 2007; Hutchinson and Turk-Browne, 2012), although the specific role of the memory system in involuntarily directing attention to previously reward-associated stimuli remains to be clarified.

Prior rodent and human studies evaluating the neural correlates of sustained fear or adaptive anxiety have identified corresponding neural activity in the dorsal amygdala, particularly in the central extended amygdala (CeA) and bed nucleus of the stria terminalis (BNST; Davis et al., 2010; Alvarez et al., 2011). However, due to the small size of these neuronal populations and the limited spatial resolution, few fMRI studies have studied these regions under conditions of experimentally-induced anxiety. In our task assessing overt attentional capture, we identified voxels activated under threat within the amygdala in the present study, but our whole-brain analyses were limited in differentiating between the neuronal populations within the amygdala. Future research using targeted, higher-resolution imaging sequences coupled with analytical techniques such as multivoxel pattern analyses (MVPAs) may provide further insight in piecing out the functional role of specific neuronal populations within amygdala in modulating anxiety in attention networks.

In the present study, we examined the neural processes by which threat reduces the distracting quality of previously reward-associated stimuli. Our neuroimaging results support the ABC model of neural processing, but show that the resulting bias in the representation of visual stimuli need not magnify distraction as measured from behavior and can even reduce it, calling for a more nuanced interpretation of the functional role of ABC in the control of visual orienting. Our study extends the concept of ABC in the brain beyond physically salient stimuli to stimuli that have elevated priority by virtue of learning history, as well as to negative arousal arising from the threat of an unpredictable and aversive biological event (as manipulated via ToS). Our findings have additional implications for the signal suppression hypothesis by demonstrating an explicit link between elevated stimulus-evoked responses in the visual system and reduced behavioral distraction, and offer novel insights into why elevated attentional priority can at times seemingly paradoxically reduce the distracting quality of stimuli (Moher et al., 2015).

## Synthesis

Reviewing Editor: Philippe Tobler, University of Zurich Decisions are customarily a result of the Reviewing Editor and the peer reviewers coming together and discussing their recommendations until a consensus is reached. When revisions are invited, a fact-based synthesis statement explaining their decision and outlining what is needed to prepare a revision will be listed below. The following reviewer(s) agreed to reveal their identity: Christoph Ploner.

Thank you for a responsive review. The only point worth considering is to include estimation statistics for figure 2 and 4.
