# Supplementary figures and images for: Arousal-Biased Competition Explains Reduced Distraction by Reward Cues under Threat
Source: eNeuro. 2020 Jul 7;7(4):ENEURO.0099-20.2020. doi: 10.1523/ENEURO.0099-20.2020 (PMC7340842; doi:10.1523/ENEURO.0099-20.2020)

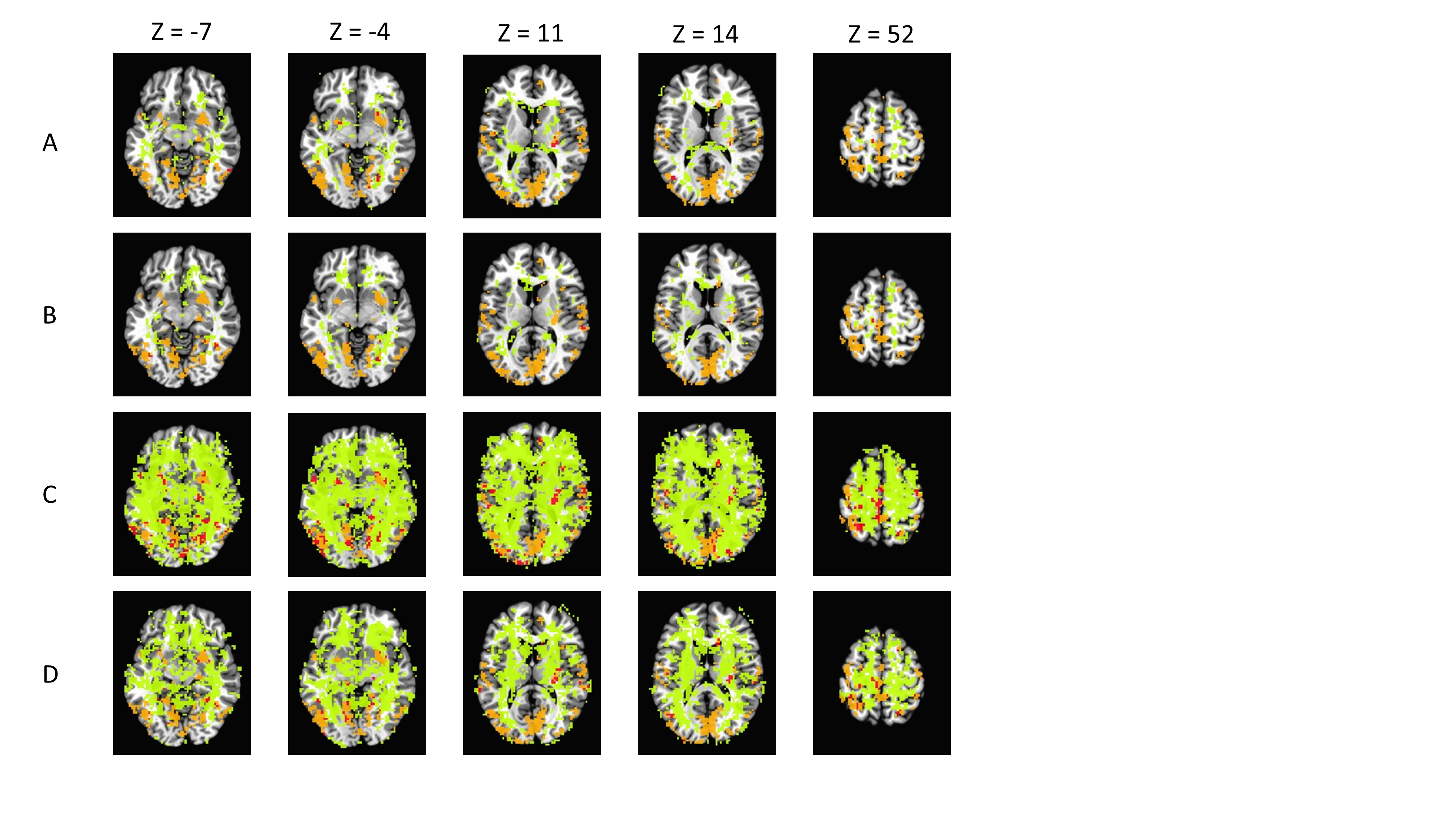

Supplement: Extended Data Figure 3-1 — Effect of distractor (green), threat (orange), and overlap between the two (red) depicted for each of the four distractor contrasts: (A) distractor on left, target on opposite side, (B) distractor on right, target on opposite side, (C) distractor on right, target on same side, and (D) distractor on left, target on same side. Note that these activation maps are shown with all subjects included, the contrasts underlying which served as the basis for the leave-one-subject-out procedure for ROI definition (with the full extent of the resulting ROIs shown across subjects in Fig. 3). Download Figure 3-1, TIF file. [file enu-eN-NWR-0099-20-s02.tif]
